# Multicentre phase II study of gemcitabine and cisplatin in malignant pleural mesothelioma

**DOI:** 10.1038/sj.bjc.6600118

**Published:** 2002-02-01

**Authors:** J M W van Haarst, P Baas, C h Manegold, J H Schouwink, J A Burgers, H G de Bruin, W J Mooi, R J van Klaveren, M J A de Jonge, J P van Meerbeeck

**Affiliations:** Rotterdam Oncological Thoracic Studygroup (ROTS), Department of Pulmonology, University Hospital Rotterdam, PO Box 5201, 3008 AE Rotterdam, The Netherlands; Netherlands Cancer Institute, Plesmanlaan 121, 1066 CX Amsterdam, The Netherlands; Thoraxklinik-Heidelberg Gmbh, Amalienstrasse 5, 69126 Heidelberg Germany; Medisch Spectrum Twente, PO Box 50000, 7500 KA Enschede, The Netherlands; Department of Radiology, University Hospital Rotterdam, PO Box 5201, 3008 AE Rotterdam, The Netherlands

**Keywords:** malignant pleural mesothelioma, cisplatin, gemcitabine, phase II study

## Abstract

Malignant pleural mesothelioma is a notoriously chemoresistant tumour. However, a recent single institution study showed an impressive activity of gemcitabine and cisplatin. Our aim is to investigate the efficacy and toxicity of a gemcitabine and cisplatin combination in selected and chemo-naive patients with histologically proven malignant pleural mesothelioma. Method: Gemcitabine 1250 mg m^−2^ was administered on day 1 and day 8 and cisplatin 80 mg m^−2^ was administered on day 1 in a 3-week cycle with a maximum of six cycles. Response and toxicity evaluations were performed according to WHO and NCIC-CTC criteria. Pathology and radiology were centrally reviewed. Results show that in 25 evaluable patients, four PR were observed (ORR 16%, 95% CI 1–31%). Responses of seven patients were unevaluable. No unexpected toxicity occurred. Time to progression was 6 months (5–7 months) with a median survival from registration of 9.6 months (95% CI 8–12 months). In conclusion this trial excludes with 90% power a response rate of greater than 30% in patients with malignant pleural mesothelioma using a combination of gemcitabine and cisplatin at the proposed dose and schedule.

*British Journal of Cancer* (2002) **86**, 342–345. DOI: 10.1038/sj/bjc/6600118 www.bjcancer.com

© 2002 The Cancer Research Campaign

## 

Malignant pleural mesothelioma (MPM) is an almost always lethal tumour. The key etiological agent is the prior inhalation of asbestos dust. Demographic exposure data indicate that its incidence is expected to further increase in the next decade in most industrialised countries. The natural history is characterized by a median survival of nine to 14 months, with less than 5% 5 year-survivors. Disease extent at diagnosis, performance status and histological subtype are the main prognostic factors (Curran *et al*, 1998; Herndon *et al*, 1998). Chemotherapy results in a less than 20% response rate and has not yet been shown to improve survival. Doxorubicin, mitomycin, cisplatin, vinorelbine and high dose methotrexate are among the drugs showing some activity. Combination chemotherapy does not consistently appear to provide better results than single agents, although response rates have been higher in some studies (Ong and Vogelzang, 1996; Baas *et al*, 1998; Steele *et al*, 2000). The combination of cisplatin and gemcitabine was reported to be synergistic in terms of cytotoxicity, both *in vitro* and *in vivo* (Peters *et al*, 1995). Used as single agents response rates of cisplatin and gemcitabine in malignant mesothelioma have been reported of 13–14% and 7–24% respectively (Mintzer *et al*, 1985; Zidar *et al*, 1988; Van Meerbeeck *et al*, 1999; Kindler *et al*, 2001). Recently, an objective response rate of 48% has been reported with the combination of cisplatin and gemcitabine in malignant mesothelioma in a single institution study (Byrne *et al*, 1999). These results prompted us to conduct a multicentre confirmation phase II study with this combination therapy.

## MATERIALS AND METHODS

Patients with histologically confirmed MPM, who had received no prior chemotherapy were accrued into this study. Tumour extension was classified according to the International Mesothelioma Interest Group (IMIG, 1995) and had to be bidimensionally measurable in at least one target lesion. Patients with just pleural effusion were not eligible. Previous intracavitary treatment was allowed, provided no cytotoxic drugs had been used. Patients had to be between 18 years and 75 years with a WHO performance status of 0 to 2, have an adequate haematological (hemoglobin >9.5 g dl^−1^, granulocyte count ⩾2×10^9^ l^−1^, platelet count ⩾100×10^9^ l^−1^), hepatic (bilirubin ⩽25 μmol l^−1^) and renal (creatinine clearance ⩾60 ml min^−1^) function. Prior surgery was permitted, as well as prior or concomitant radiotherapy of painful lesions, needle tracks or surgical scars, provided that the indicator lesions were outside the irradiated field. Patients with symptoms or signs of metastases in the central nervous system and those with a recent history of body weight loss of >10% were excluded. Written informed consent from each patient had to be obtained before patient entry. Approval by the medical ethical committees of the participating centres was obtained.

### Therapy

Gemcitabine at a dose of 1250 mg m^−2^ was diluted in normal saline and administered intravenously over 30 min on days 1 and 8 of each 21-day cycle. Cisplatin at a dose of 80 mg m^−2^ was dissolved in saline and administered intravenously in 3 h on day 1 of each cycle after the gemcitabine administration. Cisplatin infusion was preceded by parenteral administration of a 5HT-3 receptor antagonist and corticosteroids, and the infusion program contained at least 2 l fluid as hyperhydration. Blood cell counts were assessed weekly, and liver and renal functions were checked before each cycle. Treatment cycles were repeated every 21 days, provided toxic effects were not prohibitive and there was no clinical evidence of tumour progression. The dose of gemcitabine and cisplatin for subsequent cycles was adjusted according to actual weight at retreatment. Dose reductions of gemcitabine to 1000 mg m^−2^ were given in the event of febrile neutropenia, severe bleeding (NCIC grade IV), ANC nadir <0.5×10^9^ l^−1^ or a platelet nadir <50×10^9^ l^−1^ for more than 1 week, or grades 3 or 4 non-haematological toxicity (excluding nausea, vomiting and alopecia). However, in the event of an increase in serum creatinine >1.5 times the upper limit of normal, or a creatinine clearance <60 ml min^−1^, cisplatin was omitted and treatment was continued with gemcitabine only. The gemcitabine dose on day 8 of a cycle was reduced to 1000 mg m^−2^ in the case of a leukocyte count between 1–2×10^9^ l^−1^ or a platelet count between 50–100×10^9^ l^−1^. Gemcitabine was omitted on day 8 in case of a leukocyte count below 1×10^9^ l^−1^ or a platelet count below 50×10^9^ l^−1^. Treatment was continued up to six cycles, unless tumour progression, patient refusal or unacceptable toxicity developed or the investigator thought that further treatment was not in patient's benefit anymore.

### Response criteria

Tumour response was assessed with target lesions at baseline, after every second cycle and at the end of treatment, according to WHO criteria (Vantongelen, 1994). Target lesions had to be at least 2.5 cm in greatest diameter. Nodular thickening of the pleura was accepted as a target lesion if the thickening was at least 2 cm in its greatest perpendicular diameter and associated with a bidimensional lesion. CT-scans were mandatory for evaluation of intrathoracic lesions. Objective responses had to be confirmed by two measurements, at least 4 weeks apart. Toxicity was scored according to the Common Toxicity Criteria of the NCI completed by the NCIC (1998).

### Quality of life and symptom assessment

Symptom assessment was performed using a mesothelioma checklist, which comprises the most relevant items for mesothelioma of the EORTC QLQ-C30 and the EORTC QLQ-LC13 (Aaronson *et al*, 1993; Bergman *et al*, 1994). These items consisted of a four-point symptom scale for pain, dyspnea, need to rest, sleep disturbance, weakness, fatigue, pain interfering with activity and a seven-point scale for overall quality of life (QoL) and health. A higher score on the scale represents a higher intensity of the symptoms or a lower QoL and health respectively. The checklist was used as baseline and before each new cycle of chemotherapy.

### Pathology and radiology review

Patient's suitability for enrolment was determined by the pathologic report at the treating institution. Central pathology review was performed by one of the authors (WJ Mooi) in his capacity of panel member of the Netherlands Mesothelioma Panel, unless a pathologist with special expertise in MPM had already confirmed the diagnosis. Only patients with an unequivocal histological diagnosis of MPM were considered eligible. Radiological response was reviewed by an independent radiologist (HdB).

### Statistical methods

This study was planned according to the Simon (1989) one sample two stage testing procedure, having type I and type II error rates of <10% each, in order to differentiate between a response rate of 10 and 30%. Initial analysis was planned after 16 patients had been treated, and there was a further accrual to a total of 25 patients if one or more objective responses were seen in the first 16. The regimen would be considered for further evaluation if more than four objective responses were seen in evaluable patients, suggesting a true response rate of at least 30%. To compensate for ineligibility, some extra patients were included. The Kaplan–Meier method was used to estimate overall survival and time to progression of all eligible patients (Kaplan and Meier, 1958). Changes in symptoms and quality of life were evaluated using the general linear model for repeated measure analysis of variance (Kruskall Wallis *H*-test).

## RESULTS

Between April and December 1999, 32 eligible patients were included in the study from four institutes in the Netherlands and Germany. The median interval between diagnosis and inclusion in the study was 4 months (range 0–24 months). Patient and tumour characteristics are listed in Table 1

**Table 1:**
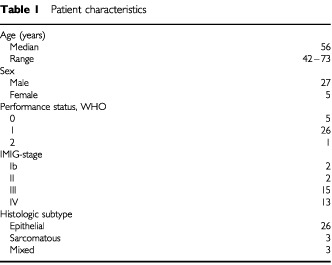
Patient characteristics

.

In total, 127 cycles were administered in 32 patients (range 1–6). In the 25 evaluable patients (120 cycles), cisplatin was administered 117 times with a mean relative dose intensity of 97% (actual administered dose per week/scheduled dose per week (calculated on body surface area)×100%). In two patients cisplatin was stopped after four cycles because of hearing loss and deterioration of creatinine clearance, respectively. Gemcitabine was administered 234 times with a mean relative dose intensity of 94%. Eight administrations were not given at day 8 of the cycle: in two patients chemotherapy was discontinued after the first part of the schedule; four administrations in two patients were omitted because of thrombocytopenia, one administration because of persisting grade 2 nausea and vomiting and one administration because of fever of unknown origin.

All eligible patients were evaluated for toxicity. The worst observed toxicity per patient is shown in Table 2

**Table 2:**
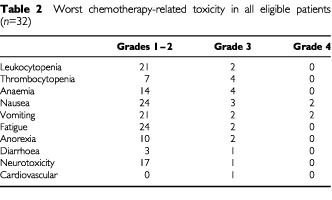
Worst chemotherapy-related toxicity in all eligible patients (*n*=32)

. The median neutrophil nadir count was 3.1×10^9^ l^−1^ and the median platelet nadir count 143×10^9^ l^−1^. In six patients, drug-related toxicity was the main reason for discontinuation of chemotherapy: four patients because of grade 3–4 nausea and vomiting, of which three also showed grade 3 fatigue, one patient because of persisting grade 2 nausea, vomiting, fatigue and pain, and one patient because of grade 3 neurotoxicity with concomitant grade 2 toxicity.

The response evaluation is summarized in Table 3

**Table 3:**
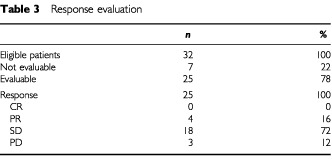
Response evaluation

. Seven patients were unevaluable for response: one patient was lost from follow up after two cycles; one patient died after one course due to a tension pneumothorax of the contralateral lung. In five instances, chemotherapy was stopped after one cycle because of toxicity in three patients, refusal in one patient, and one patient showed a rapid deterioration (dyspnea, fatigue and weight loss) presumably due to early progression and refused further therapy. All remaining 25 patients were assessable for response. Among them, we observed four partial responses (response rate 16%, 95% confidence interval (CI) 1–31%).

**Figure 1:**
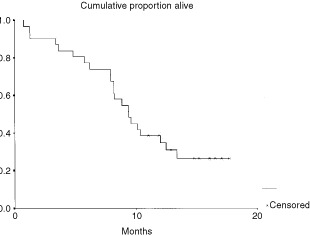
Survival from start of treatment in 32 eligible patients.

The median survival from diagnosis of the eligible patients was 14.6 months (95% CI 12.7–16.4 months) and survival from the start of treatment was 9.4 months (95% CI 7.3–11.4 months) (Figure 1

). Median time to progression calculated in 25 evaluable patients was 6.1 months (95% CI 5.7–6.5 months).

After one and two cycles of chemotherapy, QoL-data were received from 19 (76%) and 18 (72%) of the 25 evaluable patients, respectively. Compared to baseline, less pain was reported after one and two cycles of chemotherapy (*P*<0.05). There was no significant change in other symptoms or QoL items at baseline compared to during treatment (data not shown). Compliance to QoL scoring at further follow-up was below 50%. Due to this low compliance, it was impossible to make a reliable estimate of symptoms and QoL beyond two cycles of therapy.

## DISCUSSION

In this multicentre study in patients with biopsy proven MPM, we observed a 16% response rate for the treatment with gemcitabine and cisplatin. This 3-weekly schedule was given with manageable toxicity. The observed response rate is in keeping with the one reported in most cisplatin-based chemotherapy studies in malignant mesothelioma and with the ones reported in studies with cisplatin and gemcitabine as single agents (Mintzer *et al*, 1985; Zidar *et al*, 1988; Ong and Vogelzang, 1996; Van Meerbeeck *et al*, 1999; Kindler *et al*, 2001). In a single-institution study of mesothelioma, based on a four weekly regimen of cisplatin and gemcitabine in malignant mesothelioma, a response rate of 48% with a median survival of 9.5 months and an estimated 1 year survival of 41% was observed (Byrne *et al*, 1999). Recently, a response rate of 34% was reported in a multicentre study, using the same regimen (Nowak *et al*, 2000).

We have analyzed both studies in order to explain the observed differences in response rate. Of all patient characteristics, only the distribution of performance status differs significantly between both studies. Since the overall performance status was better in the present study (WHO 2: 3 *vs* 29% in the Australian study), this difference cannot explain the lower response rate in this study. In contrast to the response rate, median survival in both studies is similar and comparable to the one observed in several EORTC phase II studies (Curran *et al*, 1998), further suggesting a comparable patient selection.

As mentioned, Byrne *et al* (1999) used a 28-day cycle with gemcitabine on days 1, 8 and 15, whereas we used a 21-day cycle. The planned dose in mg m^−2^ per week of a chemotherapy cycle in these schedules, was in the present study slightly higher for both agents (cisplatin 26 *vs* 24 mg m^−2^ per week and gemcitabine 785 *vs* 619 mg m^−2^ per week). The administered dose calculated in mg m^−2^ per week was also slightly higher in the present study for both agents. In patients with non-small cell lung cancer treated with cisplatin and gemcitabine, a similar response rate with less toxicity was observed with a 3-week schedule compared to a 4-week schedule (Cardenal *et al*, 1999). So, schedule differences are not likely to explain the differences in response rate.

Both studies differ in the method to assess tumour response. In the present study, partial response was defined as a 50% reduction in the sum of the product of bidimensionally measurable lesions, whereas Byrne *et al* (1999) also included a 30% decrease in the sum of unidimensional measurements. Although both studies required a confirmed response, it is unclear whether these were independently reviewed in the Australian series. We re-evaluated the patients from one centre with the latter unidimensional criteria. From seven patients with stable disease according to the WHO criteria, we found one additional confirmed partial response with these unidimensional criteria. For rounded tumours, a 30% unidimensional reduction equals a 50% bidimensional reduction and a 65% tumour volume reduction. In mesothelioma, which often grows in the plane of the pleural surface, a unidimensional reduction in pleural thickness does not necessarily coincide with a reduction in other dimensions. If the pleural surface affected is unchanged, a unidimensional reduction of pleural thickness of 30% only reflects a 30% reduction in tumour volume. At first glance Byrne's method seems to comply with the recently introduced RECIST criteria (Therasse *et al*, 2000). However, the pleural thickness usually reflects the smallest diameter, whereas in the RECIST criteria the longest diameter is required.

A final factor that may possibly lead to differences in response rates, is a regional difference in biological behaviour of MPM or its sensitivity to an antineoplastic agent due to environmental or genetic factors (Linnainmaa *et al*, 1997). There are at present, no data to support this pharmacogenetic heterogeneity in human mesothelioma patients.

Our data suggest that QoL and symptom severity were not adversely affected by two cycles of chemotherapy and that pain control improved. The latter might either be an effect of the chemotherapy itself or reflect a more intensive pharmacological pain treatment. The value of measuring QoL in a phase II trial can be disputed. However, it allows a crude estimate with each patient being his/her own control. Symptomatic improvement has been observed more frequently than objective response in mesothelioma and other cancer patients (Steele *et al*, 2000). This has to be considered when discussing the likely palliative benefit to an individual patient. In conclusion, this trial excludes at 90% power a response rate of greater than 30% in patients with MPM using a combination treatment of cisplatin and gemcitabine at the proposed dose and schedule. Hence, from our data we cannot recommend this combination therapy as a standard therapy for MPM.
